# A Highly Sensitive Low-Temperature N-Butanol Gas Sensor Based on a Co-Doped MOF-ZnO Nanomaterial Under UV Excitation

**DOI:** 10.3390/s25144480

**Published:** 2025-07-18

**Authors:** Yinzhong Liu, Xiaoshun Wei, Yun Guo, Lingchao Wang, Hui Guo, Qingjie Wang, Yiyu Qiao, Xiaotao Zhu, Xuechun Yang, Lingli Cheng, Zheng Jiao

**Affiliations:** 1 School of Materials Science and Engineering, Shanghai University, Shanghai 200444, China; lyz2903570599@163.com (Y.L.); 13524300326@163.com (H.G.); 19370602038@163.com (Q.W.); 2250105056@shu.edu.cn (X.Z.); 2 Key Laboratory of Organic Compound Pollution Control Engineering, Ministry of Education, Shanghai University, Shanghai 200444, China; chenglingli@t.shu.edu.cn (L.C.);

**Keywords:** n-butanol, gas sensor, UV excitation, MOF-ZnO, co-doping

## Abstract

Volatile organic compounds (VOCs) are presently posing a rather considerable threat to both human health and environmental sustainability. Among these, n-butanol is commonly identified as bringing potential hazards to environmental integrity and individual health. This study presents the creation of a highly sensitive n-butanol gas sensor utilizing cobalt-doped zinc oxide (ZnO) derived from a metal–organic framework (MOF). A series of x-Co/MOF-ZnO (x = 1, 3, 5, 7 wt%) nanomaterials with varying Co ratios were generated using the homogeneous co-precipitation method and assessed for their gas-sensing performances under a low operating temperature (191 °C) and UV excitation (220 mW/cm^2^). These findings demonstrated that the 5-Co/MOF-ZnO sensor presented the highest oxygen vacancy (O_v_) concentration and the largest specific surface area (SSA), representing the optimal reactivity, selectivity, and durability for n-butanol detection. Regarding the sensor’s response to 100 ppm n-butanol under UV excitation, it achieved a value of 1259.06, 9.80 times greater than that of pure MOF-ZnO (128.56) and 2.07 times higher than that in darkness (608.38). Additionally, under UV illumination, the sensor achieved a rapid response time (11 s) and recovery rate (23 s). As a strategy to transform the functionality of ZnO-based sensors for n-butanol gas detection, this study also investigated potential possible redox reactions occurring during the detection process.

## 1. Introduction

The creation of detection tools for volatile organic compounds (VOCs) has become a crucial focus for scientists due to the fact that these gases pose serious risks to human health and environment preservation. Among these, n-butanol is extensively produced in chemical synthesis and coatings, being a colorless, transparent, and flammable compound with a distinct smell [1,2]. Extended exposure to n-butanol may result in negative health impacts such as headache, fatigue, dizziness, and vision impairment, in addition to facilitating degradation of air quality [3]. Therefore, designing n-butanol gas sensors that are simple to use, cost-effective, and highly efficient is essential for the consistent and rapid monitoring of n-butanol in the environment. Currently, numerous researchers are concentrating on designing highly effective n-butanol gas sensors. Among the various sensing technologies, gas sensors adopting metal oxide semiconductors (MOS) have emerged as the most effective, owing to their superior stability, excellent sensitivity, small size, affordable availability, and manufacture simplicity [4,5]. Common metal oxides employed in gas sensing include ZnO, CuO, SnO_2_, Fe_2_O_3_, etc. [6], as ZnO acts as the major focus in n-type metal oxide semiconductor (MOS) research and applications due to its high electron mobility (400 cm^2^·V^−1^·s^−1^), large excitation binding energy (60 meV), and wide bandwidth (3.37 eV), making it one of the most promising sensing materials [7]. Despite those advantages, there exist several disadvantages in pure ZnO gas sensors, including limited detection capability, high working temperatures, and low selectivity. Consequently, achieving precise and selective detection of target analytes in ZnO-based semiconductor gas sensors has emerged as a critical research objective in gas sensing investigation. To address these challenges, multiple strategies have been devised, involving synthesizing ZnO through different fabrication techniques, doping with rare-earth elements, modifying with noble metals, compositing with other materials, and utilizing UV excitation to enhance ZnO gas-sensing capabilities [8].

Zeolite imidazolate frameworks (ZIFs) are porous crystalline solids that have simple imidazolate ligands bridging MN_4_ tetrahedral clusters, where M denotes metal ions (Zn, Co, etc.) and N represents the nitrogen atom in the imidazolate ligand [9,10]. Because of its zeolite-like topology, large specific surface area, long-term stability, excellent crystallinity, and tunable gas adsorption capacity and inherent detection functionality, ZIF-8 has recently been identified as a promising efficient gas-sensing material [11]. ZIFs constitute a substantial subclass of metal–organic frameworks (MOFs). Metal–organic frameworks (MOFs), also termed porous coordination polymers (PCPs), are crystalline structures comprising metal centers bridged by organic ligands. When employed as sacrificial templates, MOFs can be converted into metal oxides through thermal treatment (pyrolysis/calcination). This transition can improve material conductivity while preserving the high specific surface area and outstanding adsorption capacity. Furthermore, calcination stimulates the formation of active sites while improving structural stability. As a result, metal oxide-derived MOFs become known as the particularly appealing materials for gas-sensing applications, owing to their adjustable characteristics and improved performance.

However, the monolithic composition and single-crystal-type structure of semiconducting metal oxides generated from a single MOF have limited their application in sophisticated technologies and impeded further progress. This constraint can be overcome by combining two different metal oxide semiconductors (MOSs) to considerably improve material sensitivity. The two materials combining to form the MOF, such as ZIF-8 and ZIF-67, share identical organic ligands and exhibit similar topological structures and lattice properties [12]. As a result, this bimetallic MOF derivative enables new opportunities for increasing the gas sensor operating temperatures while optimizing their sensitivity, selectivity, and reaction time. For instance, Luo et al. [13] synthesized a Co-ZnO composite material via a chemical precipitation method. By varying the Co-doping concentration, they successfully enhanced the sensor response to low-concentration volatile organic compounds (VOCs), including isopropanol, acetone, methanol, ethanol, formaldehyde, and ammonia. Among the tested materials, the 10% Co-ZnO nanoflower sensor exhibited the highest responsiveness (22.5) and outstanding endurance when exposed to 5 ppm isopropanol at 225 °C. Meanwhile, Yuan et al. [14] fabricated Co_3_O_4_/ZnO nanorods by the simple one-step hydrothermal method, enabling the successful detection of 100 ppm acetone at 250 °C. Furthermore, Zhang et al. [15] synthesized cobalt-modified zinc oxide nanosheets using the hydrothermal technique, with ZIF-67 serving as the cobalt source. The sensor displayed a response of 2540 to 100 ppm 2-butanone at 270 °C, representing a 21-fold increase compared to pure ZnO. Additionally, the sensor demonstrated an impressive detection limit of 24 ppb, making it suitable for applications requiring trace-level detection of the target gas.

Photo-excitation techniques, especially UV excitation, have garnered significant interest lately as a means of enhancing ZnO gas sensor performance. Light irradiation can produce photogenerated carriers that increase electron density in the sensor material and enhance its detection capability. Exposure to light alters the charge state of gas molecules on the material surface, lowering the crystal boundary potential barrier and improving the gas adsorption. Additionally, light can activate the target gas molecules, strengthen the chemical interaction, boost the gas desorption, promote the carrier tunneling effect, and thicken the electron depletion layer [8,16]. For example, Jiang et al. [17] developed UV-excited gas sensors based on porous nanowires sensitized with ZnO quantum dots. The optimal operating temperature for the SZQ1% sensor under UV irradiation was lowered from 120 °C to 40 °C, enabling room temperature detection of 10,000 ppb NO_2_. Simultaneously, its UV irradiation response value rose to 24.1, significantly outperforming the non-illuminated response (~3). Additionally, the SZQ1% sensor recovery time dropped from >30 min (without UV) to 470 s (with UV), while the reaction time to 1000 ppb NO_2_ was lowered from 286 s to 251 s under UV illumination. These enhancements significantly improved the sensor’s response and recovery kinetics. By facilitating the dissociation of adsorbed water, UV illumination enhanced gas sensitivity while mitigating humidity and temperature dependence. This improvement was primarily attributed to UV excitation of the SZQ composite, which produced additional photogenerated carriers. Similarly, Wang et al. [18] synthesized flower-like ZnO composites decorated with N3 in a single-step process. The findings showed that the sensor containing 0.1 wt% N_3_-loaded ZnO performed better under green light than under UV or in dark conditions when exposed to ethanol gas. This improvement stemmed from the enhanced energy utilization, broader light absorption spectrum, and higher photoelectric conversion efficiency under green light illumination. The enhanced ethanol-sensing capabilities can be ascribed to the prolonged lifetime of photogenerated carriers, the light-activated surface reactions, and facilitation of ethanol adsorption/desorption kinetics on ZnO surfaces. Furthermore, the composite structure effectively suppressed the recombination of photogenerated electron–hole. Zhang et al. [19] developed a high-performance light-assisted ethanol sensor utilizing the NiO-ZnO p-n heterojunction. The 3% Ni-ZnO achieved a detection limit of 102 ppb and tripled the ethanol response compared to pure ZnO. In contrast to dark settings, UV illumination enabled quick and reversible ethanol detection at lower temperatures (125 °C). The ZnO band gap was successfully narrowed, and the energy threshold for electron excitation was effectively lowered by the incorporation of NiO. The inter-band transitions allowed more electrons to participate in the surface reaction under light illumination and significantly improved the sensor’s overall performance.

Based on the comprehensive research described above, in this study, Co-doped MOF-derived ZnO sensors were synthesized via a straightforward precipitation method. The efficacy of these sensors for detecting n-butanol gas was assessed through the evaluation of their gas-sensing capabilities at a low operating temperature and under UV light (200 mW/cm^2^). It was demonstrated that the 5-Co/MOF-ZnO sensor achieved superior reactivity, selectivity, and stability for n-butanol gas detection compared to its pure MOF-ZnO counterpart. Furthermore, the underlying redox reactions during detection were examined to clarify the sensing mechanism. A robust strategy for effectively enhancing n-butanol gas detection using ZnO-based sensors is proposed in this work.

## 2. Experimental Section

### 2.1. Materials and Synthesis

All chemicals were procured from suppliers in China: Cobalt nitrate hexahydrate (Co(NO_3_)_2_⋅6H_2_O) with a purity of 99% was obtained from Shanghai Macklin Biochemical Technology Co., Ltd., Shanghai, China; zinc acetate dihydrate (Zn(CH_3_COO)_2_⋅2H_2_O), ethanol (C_2_H_5_OH), and methanol (CH_4_O) were obtained from Sinopharm Chemical Reagent Co., Ltd. Shanghai, China; and 2-methylimidazole (2-Mim, 98%) was obtained from Shanghai Aladdin Biochemical Technology Co., Ltd., Shanghai, Chian. All chemical reagents used in the studies were of analytical grade and used without further purification. Sensor bases and related consumables were provided by Zhengzhou Weisheng Electronic Technology Co. Ltd., Zhengzhou, China.

### 2.2. Preparation of Pure MOF-ZnO and x-Co/MOF-ZnO Materials

The results of Klaus and Michael’s investigations served as the basis for the production of MOF-ZnO nanoparticles [20]. The molar concentration ratio of Zn^2+^ to 2-Mim of 1:10 was used. Specifically, 0.48 g of Zn (CH_3_COO)_2_⋅2H_2_O was dissolved in 50 mL of methanol to create solution A, calculated as 0.043 M. Likewise, 1.78 g of 2-Mim was dissolved in 50 mL of methanol to create solution B (0.430 M). After adding solution A progressively to solution B, the mixture was agitated for 24 h to facilitate the reaction. The reaction mixture underwent vacuum filtration, yielding the precipitation of white precipitates. These precipitates were then placed in a drying oven set at 70 °C for 12 h to form the metal–organic framework (MOF) precursor, ZIF-8. The precursor was then ground into a fine powder using a mortar and subsequently calcined in a muffle furnace. The temperature was incrementally increased by 2 °C per minute until it reached 450 °C. This temperature was sustained for 2 h, before the sample was allowed to cool naturally, leading to the formation of pure MOF-ZnO.

The preparation process utilized to produce MOF-ZnO was also employed for x-Co/MOF-ZnO. The successful synthesis of a series of MOF-ZnO nanocomposites with different doping levels was carried out by changing the mass ratio of Co/(ZnO + Co) from 1 wt% to 7 wt%. The samples with Co/(ZnO + Co) mass percentage of 1 wt%, 3 wt%, 5 wt%, and 7 wt% were designated as x-Co/MOF-ZnO (1-Co/MOF-ZnO, 3-Co/MOF-ZnO, 5-Co/MOF-ZnO, and 7-Co/MOF-ZnO, respectively).

**Figure 1:**
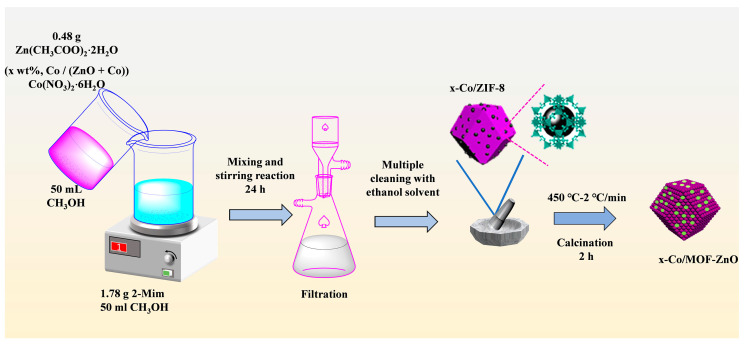
Schematic representation of the synthetic procedure.

The entire preparation process is illustrated in detail in Figure 1.

### 2.3. Characterization

The physical phase composition and crystal structure were analyzed using the D/MAX 18 kV microcomputer-controlled X-ray diffractometer (XRD) with Cu Ka1 radiation (λ = 1.5406 Å) over the 10°–90° scanning range. The elemental composition was determined by X-ray photoelectron spectroscopy (XPS, ESCALAB 250Xl, Al Ka source, Thermo Fisher Scientific, Waltham, MA, USA). The surface morphology and microstructure were characterized using an environmental scanning electron microscope (SEM, JEOL Ltd., Tokyo, Japan) and high-resolution transmission electron microscope (HRTEM, JEM-2100F, JEOL Ltd., Akishima, Japan). The chemical composition was assessed via the energy-dispersive X-ray spectrometer (EDS, JEOL Ltd., Tokyo, Japan) installed in the SEM unit. Nitrogen adsorption–desorption isotherms were measured using the automatic surface analyzer (Autosorb-IQ, Quantachrome Instruments, Boynton Beach, FL, USA). The Brunauer–Emmett–Teller (BET) and Barrett–Joyner–Halenda (BJH) approaches were applied to obtain the surface area and pore size distribution, respectively. Ultraviolet–visible diffuse reflectance spectroscopy (UV, UV-3600, Shimadzu Corporation, Kyoto, Japan) was performed in the 200–800 nm wavelength range.

### 2.4. Fabrication and Performance Testing of Gas Sensors

**Figure 2:**
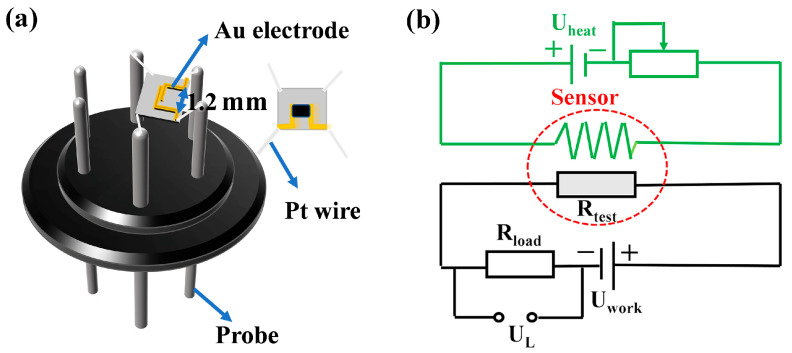
(**a**) A schematic representation of the gas sensor and (**b**) the circuit diagram utilized for the gas sensing experiment.

Previous reports on the issue were cited in the construction procedure for this sensor [8]. Five milligrams (5 mg) of the gas-sensitive material was mixed in a drop of turpentine before being uniformly applied to a ceramic chip (3 × 3 × 0.25 mm) featuring printed gold electrodes. The chip was connected to the sensor base via four Pt wires at its corners. Structural diagrams of the chip sensor are presented in Figure 2a. The assembly was dried in a vacuum drying oven set at 60 °C for 2 h to remove residual turpentine. The dried sensor was then mounted on the circuit board and aged in dedicated equipment (TS64, Zhengzhou Weishen Electronic Technology Co., Ltd., Zhengzhou, China) at a heating voltage of 3.37 V for three days to activate the sensor and enhance stability and activate sensing properties The sensing capabilities were evaluated using the WS-30A Gas Sensor Measurement System (Zhengzhou Weishen Electronic Technology Co., Ltd., Zhengzhou, China). The data were gathered and examined with dedicated software. The operating voltage was maintained at 5.0 V, while the working temperature was regulated through heating voltage control. The voltage in the test circuit was carefully regulated using a load resistance card. Figure 2b illustrates the gas sensing circuit diagram employed in the gas sensing experiment. For the entirety of the tests, the static gas distribution method [21] was employed, with the ambient air humidity maintained at approximately 20% and the temperature within the test chamber recorded at approximately 25 °C.

In this paper, the response of the gas sensor was defined as

(1) $$\mathrm{Response}=R_{a}/R_{g}$$

where Ra denotes the sensor resistance in air, and Rg represents the resistance in the target gas [8]. For n-type semiconductors, the response time (τres) and recovery time (τrec) are defined as the duration required for the sensor resistance to stabilize at 90% of its initial value after gas adsorption and desorption [22]. All gas sensing tests were initially performed in a dark environment. Subsequently, measurements were repeated under ultraviolet (UV) light illumination.

## 3. Results and Discussion

### 3.1. Structure and Morphology Analysis

**Figure 3:**
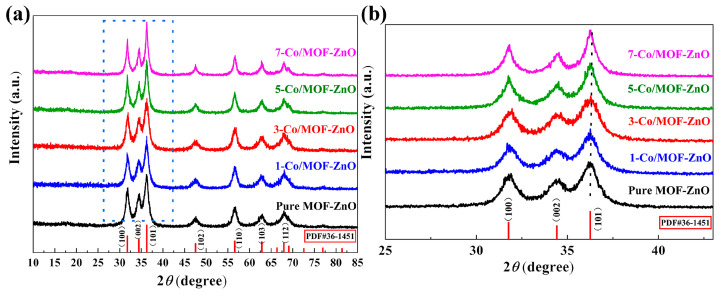
X-ray diffractometer patterns of pure MOF-ZnO and x-Co/MOF-ZnO material series with 2θ: (**a**) 15°–85°; (**b**) 25°–43°.

Figure 3 shows the XRD patterns for pure MOF-ZnO and x-Co/MOF-ZnO nanoparticles. All diffraction peaks matched well with the typical hexagonal wurtzite structure of ZnO, as referenced in JCPDS No. 36-1451. In Figure 3a, the Co-doped MOF-ZnO samples present no additional diffraction peaks indicating the absence of Co-related compounds (such as Co, CoO, or Co_3_O_4_). This suggested that the Co element was uniformly incorporated into the ZnO lattice without crystallizing into different phases [23]. Compared to pure MOF-ZnO, Co-doped MOF-ZnO exhibited a higher diffraction peak intensity. Figure 3b shows a shift of approximately 0.12° in 2θ between MOF-ZnO and 5-Co/MOF-ZnO. These findings proved partial substitution of Zn ions by Co ions in the lattice, as the radius of Co ions (0.058 nm) was smaller than that of Zn ions (0.074 nm). When the Co-doping ratio was less than 5 wt%, this substitution made the lattice contraction shrink, slightly shifting diffraction peaks towards higher angles. Furthermore, the angular displacement grew with higher Co concentrations in the precursor [24]. Nevertheless, when the Co-doping ratio approaches 5 wt%, the excess Co doped only adsorbs on the ZnO surface rather than fully entering the ZnO lattice, increasing the lattice parameter and shifting the diffraction peak to a lower angle.

Additionally, based on the most intense (101) diffraction peak, the average crystallite sizes of Co/MOF-ZnO and unmodified MOF-ZnO were calculated by employing Scherrer’s formula applied to the Full Width at Half Maximum (FWHM), as displayed in Table 1 [25].

(2) D=Kλ/βcosθ

The wavelength of CuKα1 radiation and the FWHM of the (101) peak are denoted by λ and β, respectively, whereas K is a constant (0.89), and θ is the angle of Bragg diffraction.

The dual doping effect of Co^2+^ on crystal development was evidently presented. Since the ionic radius of Co^2+^ is smaller than that of Zn^2+^, low-concentration doping below 5% resulted in a suppressed size due to lattice strain, followed by an enlarged size driven by the enhanced nucleation kinetics at higher concentrations.

**Figure 4:**
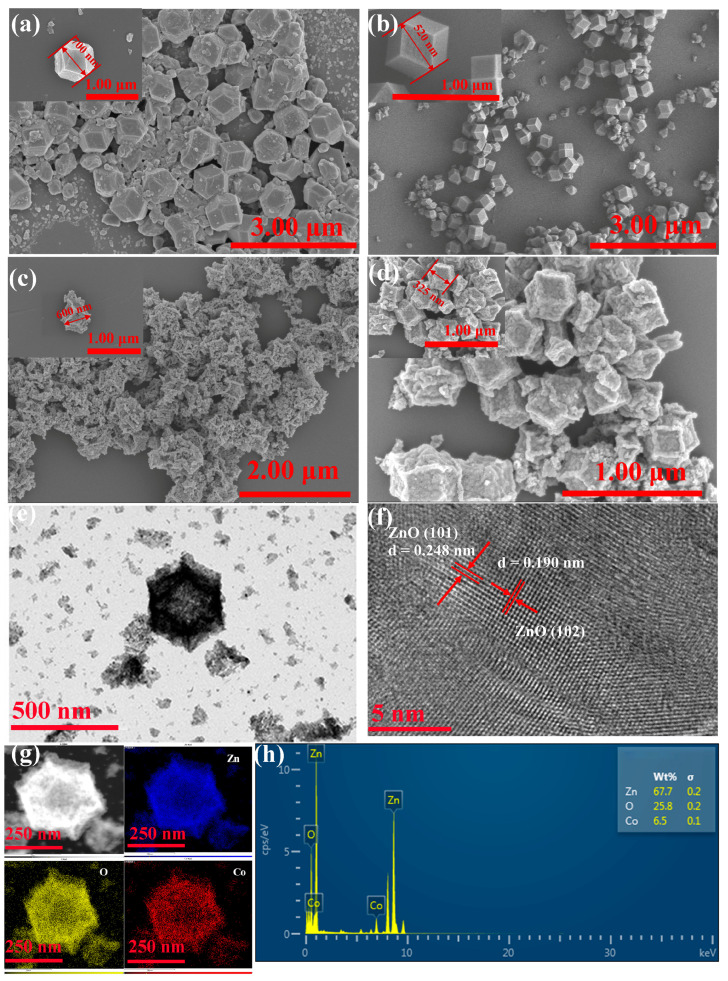
Scanning electron microscope images: (**a**) ZIF-8, (**b**) 5-Co/ZIF-8, (**c**) pure MOF-ZnO, and (**d**) 5-Co/MOF-ZnO. Transmission electron microscope and high-resolution transmission electron microscope images of 5-Co/MOF-ZnO are also shown (**e**,**f**), along with the EDS analysis results of 5-Co/MOF-ZnO (**g**,**h**).

SEM and TEM analyses were performed on ZIF-8 and 5-Co/ZIF-8 before and after calcination to explore their morphological evolution, as illustrated in Figure 4. The as-synthesized pristine ZIF-8 particles exhibited a homogeneous dodecahedral microstructure (Figure 4a) with an average particle size of ~700 nm. For 5-Co/ZIF-8 (Figure 4b), lattice distortion induced by the smaller ionic radius of Co^2+^ (vs. Zn^2+^) reduced the average particle size to ~520 nm, while maintaining the dodecahedral morphology, Notably, Co-doping produced smoother and more defined prismatic dodecahedral. Figure 4c,d displays the morphologies of the derived ZnO nanoparticles (from ZIF-8 and 5-Co/ZIF-8, respectively). The calcined samples structurally contracted and partially collapsed, resulting in particle size shrinkage. The ligand pyrolysis and the framework shrinkage during thermal treatment could be attributed to this phenomenon [26].

The nanostructural characteristics of 5-Co/MOF-ZnO were subjected to further investigation via TEM. As depicted in Figure 4e,f, the calcined sample exhibited structural collapse at particle margins and reduced grain size, consistent with SEM observations. High-resolution transmission electron microscopy (HRTEM) images identified distinct (101) lattice fringes of MOF-ZnO with a d-spacing value of 0.247 nm. The elemental distribution analysis in the 5-Co/MOF-ZnO particles was performed by energy-dispersive X-ray spectroscopy (EDS) (Figure 4g,h), and we identified Zn, O, and Co as the main constituents with the uniform distribution. Quantitative EDS results confirmed the effective nanoparticle synthesis of 5-Co/MOF-ZnO, as evidenced by the Zn (67.7 wt%), O (25.8 wt%), and Co (6.5 wt%). Due to the presence of local enrichment and the semi-quantitative character of EDS analysis, the Co content as determined by EDS is marginally higher than the nominal value.

### 3.2. Surface and Bandwidth Analysis

**Figure 5:**
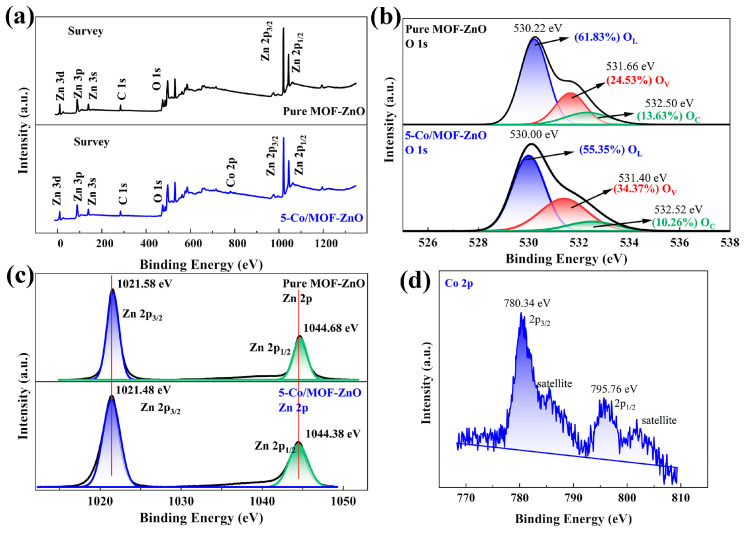
X-ray photoelectron spectroscopy spectra of pure MOF-ZnO and 5-Co/MOF-ZnO: (**a**) survey, (**b**) O 1s, (**c**) Zn 2p, and (**d**) Co 2p.

The surface composition and valence states of each element in the pure MOF-ZnO and 5-Co/MOF-ZnO microstructures were further examined using X-ray photoelectron spectroscopy (XPS) analysis, and the results are revealed in Figure 5. The findings indicated the Zn, O, and Co elements’ coexistence with 5-Co/MOF-ZnO, consistent with the EDS results and confirming the effective doping of Co into MOF-ZnO (Figure 5a). As the calibration element, carbon did not show any discernible impurity peaks. A notable disparity in ionic radii between Co and Zn ions was caused by the introduced defects in the 5-Co/MOF-ZnO crystal structure, specifically promoting the creation of oxygen vacancies [14]. Figure 5b reveals the high-resolution O 1s spectra of the pure MOF-ZnO and 5-Co/MOF-ZnO samples. Subsequent peak-fitting analysis revealed three distinct components: surface chemisorbed oxygen (O_C_), oxygen vacancy (O_V_), and lattice oxygen (O_L_). The dominant peaks disclosed at 530.21 eV and 530.00 eV for pure MOF-ZnO and 5-Co/MOF-ZnO, respectively, were indicative of the formation of Zn-O bonding by lattice oxygen ions (O^2−^), corresponding to the zincite structure [27]. Furthermore, the O_V_ concentrations for pure MOF-ZnO and 5-Co/MOF-ZnO were 24.53% and 34.37%, respectively, suggestive of O_V_ enrichment by the addition of Co-dopant into MOF-ZnO. This enhancement in oxygen vacancies would be contributed to the higher availability of surface active sites, in turn facilitating the generation of negatively charged oxygen species on the surface. While some studies report that the photoelectron signal at 531–532 eV originates exclusively from oxygen-containing compounds (e.g., surface hydroxyl groups and chemisorbed water) rather than oxygen vacancies [28,29], we note a critical indirect relationship. Specifically, oxygen vacancies substantially promote the formation of these surface species through reactive processes. This causal link establishes a well-documented positive correlation between the 531–532 eV signal intensity and oxygen vacancy concentration. These adjustments accelerated the sensing reactivity to the target gas and thus improved the gas-sensing capability [30].

High-resolution XPS spectra of Zn 2p in 5-Co/MOF-ZnO and pure MOF-ZnO are displayed in Figure 5c. For pure MOF-ZnO, the Zn 2p_3_/_2_ and Zn 2p_1_/_2_ binding energies were 1021.58 eV and 1044.68 eV, respectively, indicating Zn^2+^ species [31]. In contrast, the corresponding binding energies for 5-Co/MOF-ZnO were 1021.48 eV and 1044.38 eV, respectively, showing a slight decrease in comparison to MOF-ZnO, this shift was attributed to the interaction and electron transfer between Co and MOF-ZnO [14,32]. Additionally, the incorporation of Co into the MOF-ZnO lattice influenced the Zn 2p binding energy. Figure 5d shows the Co 2p high-resolution spectrum, as the peaks at 780.07 eV and 795.84 eV assigned to Co 2p_3_/_2_ and 2p_1_/_2_ energy levels, respectively, corresponded to the binding energy of Co 2p in Co_3_O_4_, which suggested that Co was presented primarily as Co^2+^ and Co^3+^ ions and effectively doped into the lattice [14]. The satellite peaks observed at approximately 785.42 eV and 801.73 eV are also characteristic of Co ions [33].

**Figure 6:**
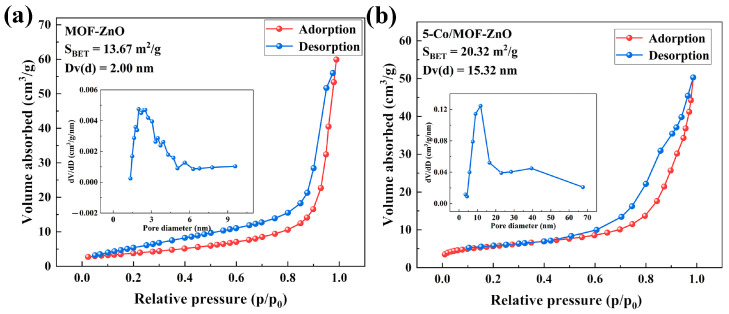
Pore size distributions (inset) and nitrogen adsorption–desorption isotherms: (**a**) MOF-ZnO and (**b**) 5-Co/MOF-ZnO.

The characteristics of the gas surface produced significantly influenced the material gas-sensitive properties. Nitrogen adsorption–desorption isotherms were used to determine the surface area and average pore diameters of pure MOF-ZnO and 5-Co/MOF-ZnO, revealing the impact of Co-doping (Figure 6a,b). The adsorption isotherms of both samples were categorized using Brunauer’s categorization as ‘Type IV’ with H3-type hysteresis loops [34]. The specific surface area increased from 13.67 m^2^/g for pure MOF-ZnO to 20.32 m^2^/g for 5-Co/MOF-ZnO, while the average pore size expanded from 2 nm to 15.32 nm. SEM analysis confirmed similar morphologies for both materials. 5-Co/MOF-ZnO’s bigger pore size and increased surface area can be explained by the Co element’s grain growth-limiting action, which encouraged the production of smaller but more frequent crystallites in the finished product; by creating more interstitial spaces during particle aggregation, these finer grains eventually produced more porous structures with higher specific surface area and pore volume. The increased specific surface area was attributed to the presence of Co on the material surface, demonstrating that Co-doping was beneficial for enhancing sensing capabilities for target gases.

**Figure 7:**
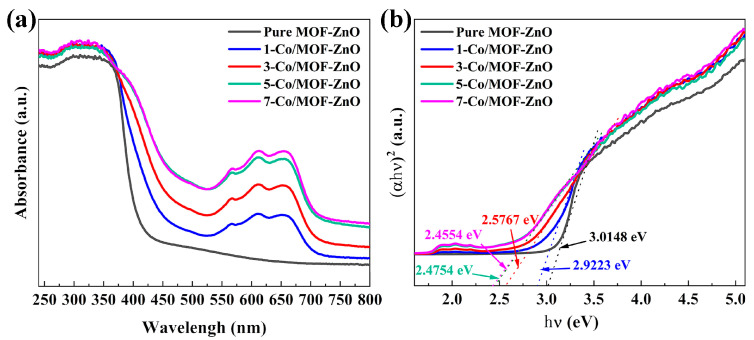
(**a**) Ultraviolet-visible absorption spectra, (**b**) forbidden bandwidths of pure MOF-ZnO and x-Co/MOF-ZnO (x = 1, 3, 5, 7) series composites.

The optical characteristics of pure MOF-ZnO and Co-MOF-ZnO nanoparticles were assessed via the application of UV spectrophotometry (Figure 7a). Distinct absorption bands were observed around 375 nm for both materials. As the Co-doping ratio increased, the absorption band edge shifted from the ultraviolet range toward the longer wavelength direction. This redshift may be attributed to either Co-doping-induced crystal deformation or spin-related excitation generation [35].

The band gap energy, the crucial electrical property of the semiconductor nanomaterial, was calculated from the intercept of the linear portion [36]. Analysis of energy spectra utilizing the Kubelka–Munk equation facilitated the determination of the band gap; the calculated band gap energy values for pure MOF-ZnO and Co-doped MOF-ZnO series were calculated to be 3.0148 eV, 2.9223 eV, 2.5767 eV, 2.4754 eV, and 2.4554 eV, respectively (Figure 7b). The band gap of the Co-doped MOF-ZnO progressively narrowed as the Co-doping ratio increased. This band gap narrowing was responsible for the thickening of the electron depletion layer and the elevation in the number of electrons within the conduction band. These effects facilitate the movement of electrons from the valence band to the conduction band, thereby effectively improving the gas-sensing capabilities of the Co-doped MOF-ZnO.

### 3.3. Gas-Sensing Performances

**Figure 8:**
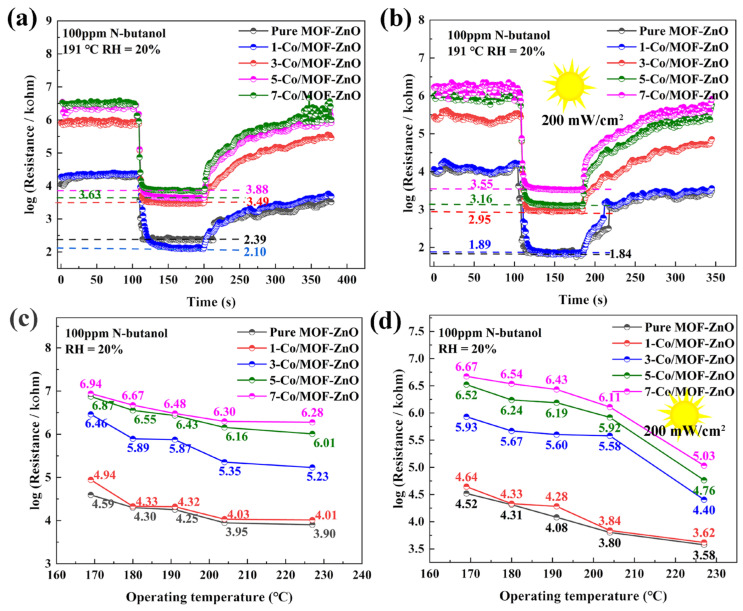
Response–recovery curves for 100 ppm n-butanol: (**a**) pure MOF-ZnO and x-Co/MOF-ZnO sensor series and (**b**) pure MOF-ZnO and x-Co/MOF-ZnO sensor series under UV light. The baseline resistance in air at various operating temperatures: (**c**) pure MOF-ZnO and x-Co/MOF-ZnO sensor series and (**d**) pure MOF-ZnO and x-Co/MOF-ZnO sensor series under UV light.

The gas-sensing properties of pure MOF-ZnO and x-Co/MOF-ZnO (x = 1, 3, 5, 7) material series were evaluated using a WS-30A system with n-butanol gas in a 20% relative humidity environment. The UV excitation was provided by a 365 nm LED-UV curing lamp (Zhongshan Purple UV Curing Lighting Appliance Factory, Zhongshan, China). The adsorption and desorption of gas molecules on the oxide surface influenced the sensor sensitivity, making operating temperature the critical factor in determining the response conditions [37]. The transient induction curves of pure MOF-ZnO and x-Co/MOF-ZnO (x = 1, 3, 5, 7) for 100 ppm n-butanol under dark and UV illumination conditions are shown in Figure 8a,b. The figures reveal that all sensors exhibit a gradual decrease in resistance upon exposure to n-butanol gas, stabilize, and then gradually recover to their baseline resistance in air after gas removal. This resistance variation aligns with n-type semiconductor behavior [38]. Additionally, it can be observed that under UV light excitation, the sensor’s baseline resistance decreases. This phenomenon occurs because UV illumination generates photoexcited holes that are captured by adsorbed oxygen via surface electron–hole recombination, while simultaneously enhancing the lifetime of unpaired photoexcited electrons. These electrons improve electrical conductivity, consequently reducing the baseline resistance [39].

Figure 8c demonstrates the semiconductor characteristics further, showing decreased resistance for all sensors as operating temperature increases. Additionally, sensor resistance steadily increased with higher Co-doping concentrations. The electron-rich Co-dopant provides additional active sites, enhancing oxygen adsorption and significantly increasing resistance. Figure 8d shows lower sensor resistance under UV illumination compared to dark conditions, indicating that UV light generates substantial photo-induced charge carriers.

**Figure 9:**
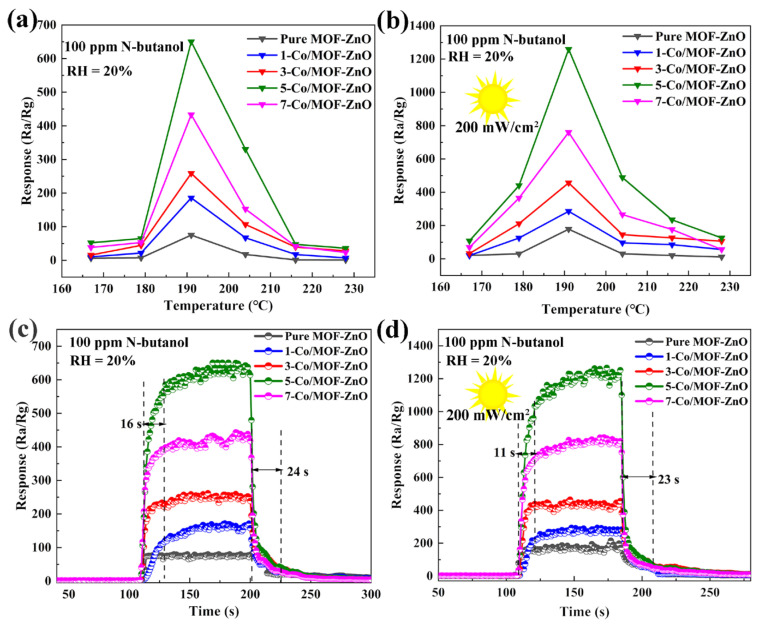
Response to 100 ppm n-butanol at different working temperatures for pure MOF-ZnO and x-Co/MOF-ZnO sensor series: (**a**) in the dark and (**b**) under UV light. This figure also shows their response–recovery profiles at 191 °C both (**c**) in the dark and (**d**) under UV.

The gas-sensing responses of pure MOF-ZnO and x-Co/MOF-ZnO (x = 1, 3, 5, 7) sensors series were assessed at 100 ppm of n-butanol gas across the temperature range of 170–215 °C, both in the dark and under UV illumination (200 mW/cm^2^), and the results are depicted in Figure 9a,b. The sensor response values first grew as the temperature rose, reaching a peak at 191 °C, and then decreased. Among these, 5-Co/MOF-ZnO achieved the maximum response value (1259.06) under UV illumination. The reduction in sensing response at lower operating temperatures may be caused by the insufficient activation energy, which prevented the effective adsorption of gas molecules onto the MOF-ZnO material. Conversely, the sensor response decrease at higher temperatures may occur because gas molecules desorb from the sensing surface more quickly than they adsorb, even with increased activation energy.

The real-time response curves to 100 ppm of n-butanol at the ideal temperature of 191 °C based on MOF-ZnO and x-Co/MOF-ZnO (x = 1, 3, 5, 7) sensor series are displayed in Figure 9c,d. The reaction value of the 5-Co/MOF-ZnO sensor in the dark reached 608.38, which was eight times greater than that of the MOF-ZnO (75.15). The MOF-ZnO sensor response value under UV illumination was 128.56, while the 5-Co/MOF-ZnO sample achieved a value of 1259.06. This represented a significant enhancement of 9.8 times compared to the MOF-ZnO sensor under UV light. Moreover, when subjected to UV light, the 5-Co/MOF-ZnO response was twice as high as that for the darkness case. UV illumination drived this improvement by enhancing the production of electron–hole pairs, which in turn facilitated electron flow and raised gas-sensing efficiency. These findings confirmed that the synergistic effect of Co-doping and UV illumination significantly advanced the material’s gas-sensing capacity [19,40]. Additionally, the response and recovery times of these sensors were evaluated at 191 °C; the 5-Co/MOF-ZnO exhibited response and recovery times of 16 s and 24 s in darkness, respectively. These decreased to 11 s and 23 s under UV illumination. Additional energy was supplied by the UV illumination, promoting the adsorption and desorption of n-butanol on the material surface, facilitating the ionization of oxygen adsorbates into oxygen ions (O^2−^, O^2−^, O^−^), leading to a faster reaction and recovery process [41].

**Figure 10:**
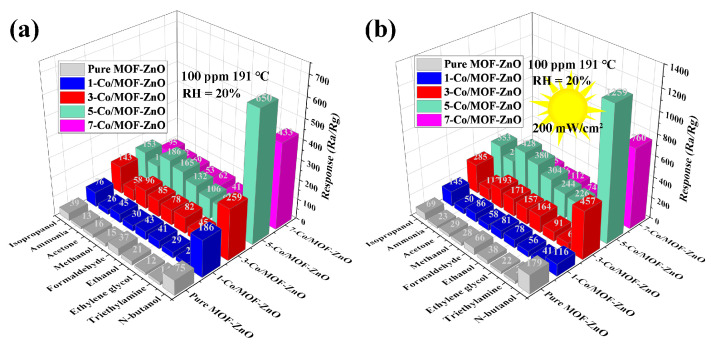
Selectivity of pure MOF-ZnO and 5-Co/MOF-ZnO sensor series for various gases below 100 ppm: (**a**) results for experiment conducted in the dark and (**b**) results for experiment conducted under UV light illumination.

Sensor selectivity is a crucial factor in determining suitability for applications in complex and dynamic environments. This research assessed the selectivity of pure MOF-ZnO and 5-Co/MOF-ZnO sensors under both dark and UV illumination conditions. Nine distinct gases (n-butanol, triethylamine, ethylene glycol, ethanol, formaldehyde, acetone, ammonia, and isopropanol), each used at a concentration of 100 ppm, were examined to evaluate sensor response (Figure 10). Figure 10a illustrates that the maximum response value of the 5-Co/MOF-ZnO sensor was 608.38 under dark conditions. In darkness, the sensor response to n-butanol was 9.50, 6.62, 6.12, 4.92, 3.93, 3.49, 5.50, and 4.24 times greater than its response to the other eight gases, respectively. Under UV illumination, the 5-Co/MOF-ZnO sensor’s response to n-butanol (1259.06) was 8.00, 5.60, 5.20, 4.10, 3.30, 2.90, 4.60, and 3.58 times greater than its responses to the other eight gases, respectively. The 5-Co/MOF-ZnO sensing material surface demonstrated the strongest n-butanol selectivity, while the response values for other reducing gases were relatively lower. The enhanced selectivity may contribute to improving the sensor’s sensitivity to n-butanol. Chemical bond breakage and alkyl chain length in gas molecules can significantly affect their reaction with semiconductor materials [42]. UV illumination could generate more photogenerated holes and electrons, accelerating the surface redox process. Cobalt incorporation lowered activation energy, enriched active sites for gas adsorption and surface reactions, and boosted the reaction rate. Furthermore, the interaction between Co and Zn promoted target molecule adsorption, further enhancing the surface reaction rate and consequently improving the sensor’s selectivity and sensitivity [43].

The gas-sensitive properties of the 5-Co/MOF-ZnO material were specifically compared with those of previously reported MOF-ZnO-based sensors (Table 2). This comparison affirmed that the 5-Co/MOF-ZnO sensor proposed in this study demonstrated significantly superior performance in detecting n-butanol.

**Figure 11:**
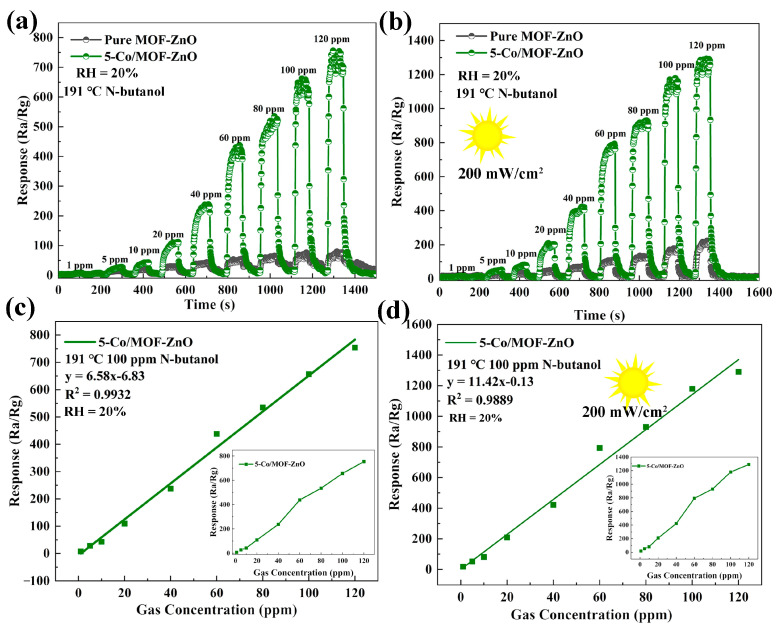
Dynamic response curves for pure MOF-ZnO and 5-Co/MOF-ZnO to varying n-butanol concentrations: (**a**) results for experiments conducted in the darkness and (**b**) results for experiments conducted under UV light. Fitted curves of MOF-ZnO and 5-Co /MOF-ZnO at distinct n-butanol concentrations: (**c**) results for experiments conducted in the darkness and (**d**) results for experiments conducted under UV light.

The sensitivity of the pure MOF-ZnO and 5-Co/MOF-ZnO sensors to varying n-butanol concentrations (10–100 ppm) was assessed at an optimal operating temperature of 191 °C under both dark conditions and UV illumination. As n-butanol concentrations ascended, the sensor response presented a linear ascension without reaching saturation, as demonstrated in Figure 11a,b. To determine detection limits and elucidate the relationship between the sensor response and n-butanol concentration, linear regression was applied for data analysis.

Figure 11c,d displays the fitted correlation coefficients (R^2^) under both darkness and UV irradiation conditions (while the insets remain unfitted). These coefficients manifested a strong linear correlation between the concentration of n-butanol and the corresponding sensor response. With respect to detecting n-butanol, the 5-Co/MOF-ZnO sensor demonstrated detection capabilities of 2.266 ppm in darkness and 1.435 ppm under exposure to UV light. These low theoretical detection limits confirm the sensor’s effectiveness regarding trace-level n-butanol detection.

**Figure 12:**
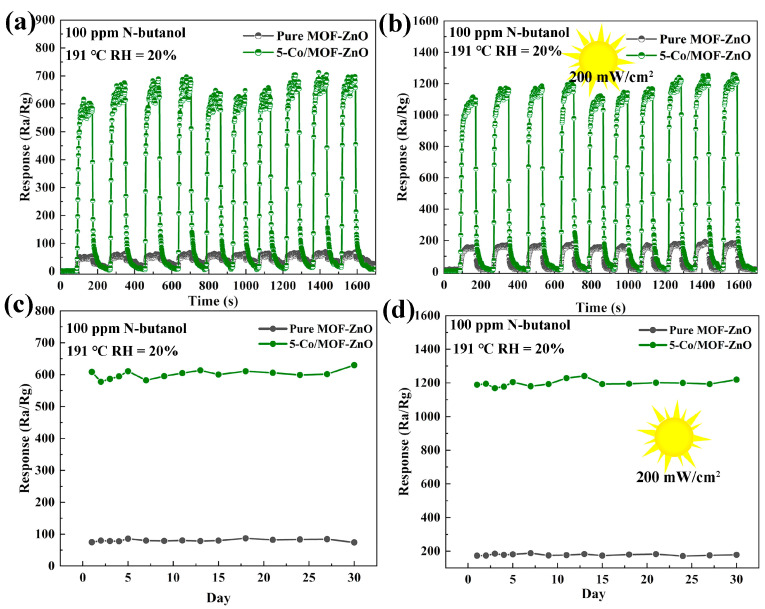
The pure MOF-ZnO and 5-Co/MOF-ZnO: Cycles of response and recovery at 191 °C and 100 ppm n-butanol: (**a**) in the dark and (**b**) under UV light illumination; Testing pure MOF-ZnO and 5-Co/MOF-ZnO sensors for long-term stability over a month in two conditions: (**c**) in the darkness and (**d**) under UV light illumination.

Cyclic stability is an essential sensor performance metric, indicating the consistency of sensor response upon repeated exposure to the target gas at the same concentration. Figure 12a,b depicts the recovery curves and dynamic response results of the pure MOF-ZnO and 5-Co/MOF-ZnO sensors to 100 ppm n-butanol at 191 °C under UV illumination and dark conditions. Both sensors showed great repeatability over ten consecutive cycles. Under UV irradiation, the 5-Co/MOF-ZnO and MOF-ZnO sensors exhibited significantly higher responses to n-butanol compared to their performance in darkness, with the MOF-ZnO sensor showing a slightly lower response. This improved reactivity to UV light can be ascribed to the efficient generation of electron–hole pairs, which raised carrier density and promoted greater participation of charge carriers to participate in the gas adsorption and oxidation processes, thereby enhancing the sensor’s response performance. Furthermore, Co-doping minimized the recombination of photogenerated electron–hole pairs. Co-doping enhanced redox processes and prolonged carrier lifetimes in the sensing material [48].

### 3.4. Gas-Sensing Mechanism

The results of 30-day measurements of 100 ppm n-butanol using pure MOF-ZnO and 5-Co/MOF-ZnO sensors at an ideal temperature of 191 °C under darkness and UV illumination are shown in Figure 12c,d. The minimal variations in response values over a month resulted in no discernible deterioration in the sensor response, demonstrating the exceptional long-term stability of both sensors, which are capable of operating reliably and consistently over prolonged periods.

**Figure 13:**
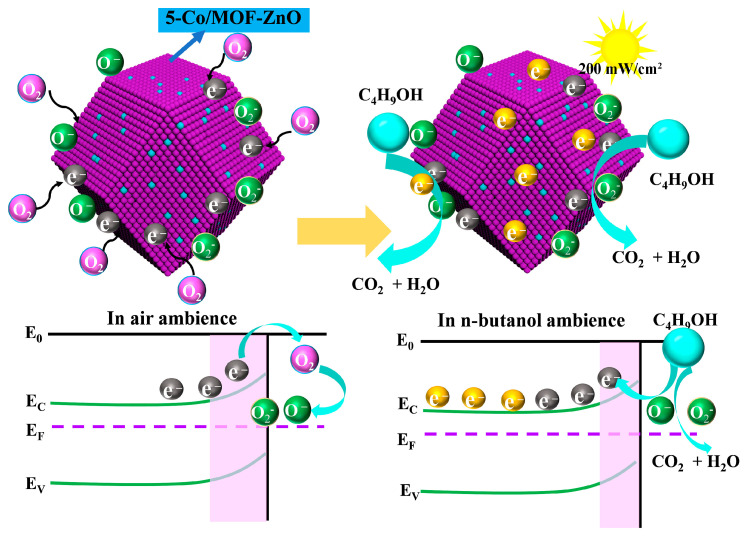
Schematic illustration depicting the sensing mechanism of the 5-Co/MOF-ZnO sensor.

The gas-sensing mechanism of these materials can be primarily attributed to the changes in electrical resistance resulting from the electron transfer between the target gas and the oxygen ions adsorbed on the surface of the sensing material [49]. As depicted in Figure 13, the exposure of the sensing material to atmospheric conditions led to the adsorption of oxygen molecules onto the surface of ZnO. This interaction facilitated the extraction of electrons from the conduction band, forming oxygen anions (O_2_^−^, O^−^, and O^2−^) [22]. The type of oxygen anion formed was predominantly determined by the sensor operating temperature. O^2−^ dominated at lower temperatures, while O^−^ and O^2−^ become progressively dominant at higher temperatures, with O^−^ being predominant at the operating temperature used in this study [22]. The reaction equations are as follows:

(3) $$O_{2}\;\left(\mathrm{gas}\right)\;\to {\;O}_{2}\;\left(\mathrm{ads}\right)$$

(4) $$O_{2}\;\left(\mathrm{ads}\right)+e^{-}\;\to {\;O}_{2}^{-}\;\left(\mathrm{ads}\right)\left(T<100{}^{\circ}C\right)$$

(5) $$O_{2}^{-}\;\left(\mathrm{ads}\right)+{\;e}^{-}\;\to \;2O^{-}\;\left(\mathrm{ads}\right)\left(100{}^{\circ}C<T<300{}^{\circ}C\right)$$

(6) $$O^{-}\;\left(\mathrm{ads}\right)+e^{-}\;\to {\;O}^{2-}\;\left(\mathrm{ads}\right)\left(T>300{}^{\circ}C\right)$$

Overall, 191 °C was identified as the ideal operating temperature for the 5-Co/MOF-ZnO sensor, since, at this temperature, oxygen ions (primarily O^−^) adsorbed onto the sensor surface. This reduces the number of electrons in the conduction band, forming a low-conductivity electron depletion layer near the surface. Consequently, the potential barrier increases, raising the sensor resistance. When n-butanol was introduced, it reacted with surface O^−^ ions on the 5-Co/MOF-ZnO material to produce CO_2_ and H_2_O. This reaction released the electrons back into the conduction band, reducing the thickness of the electron depletion layer and thereby decreasing resistance [22,50]. The following reactions summarize this process:

(7) $$C_{4}H_{9}\mathrm{OH}\;\left(\mathrm{gas}\right)\;\to {\;C}_{4}H_{9}\mathrm{OH}\;\left(\mathrm{ads}\right)$$

(8) $$C_{4}H_{9}\mathrm{OH}\;\left(\mathrm{ads}\right){+12O}^{-}\;\left(\mathrm{ads}\right)\;\to \;4CO_{2}+5H_{2}O+12e^{-}$$

Moreover, under UV illumination, 5-Co/MOF-ZnO could generate photogenerated electron–hole pairs by absorbing photon energy. These charge carriers migrate to the material surface, where holes (h^+^) oxidize adsorbed oxygen species (e.g., O^−^), releasing rapped electrons back into the conduction band. This process enhanced surface reactivity, reduced the electron depletion layer thickness, and significantly increased conductivity (decreasing resistance) [43]. The underlying reactions are summarized as follows:

(9) $$ZnO+{\mathrm{hv}\;\to \;h}^{+}\;(\mathrm{hv})+{\;e}^{-}\left(\mathrm{hv}\right)$$

(10) $$2h^{+}\left(hv\right)+2O^{-}\left(ads\right)\to O_{2}\left(g\right)$$

(11) $$O_{2}\;(g){+2e}^{-}\left(\mathrm{hv}\right)\;\to 2O^{-}\;\left(\mathrm{hv}\right)$$

(12) $$C_{4}H_{9}\mathrm{OH}\;\left(\mathrm{ads}\right)+{12O}^{-}\;\left(\mathrm{hv}\right)\;\to \;4CO_{2}+{5H}_{2}O+{12e}^{-}$$

UV illumination enhanced the performance of 5-Co/MOF-ZnO by increasing the carrier density and increasing the depletion layer. Concurrently, Co-doping optimized surface redox processes and extended carrier lifetime through efficient electron–hole separation, significantly reducing recombination losses.

## 4. Conclusions

Co-doped MOF-ZnO nanomaterials were effectively created in this study for application in n-butanol gas sensors. The experimental results confirmed that UV illumination and Co-doping greatly enhanced the sensors’ sensitivity and selectivity. The 5-Co/MOF-ZnO sensor achieved outstanding cyclic and long-term stability, alongside a significant response value of 1259.06 to 100 ppm n-butanol at an operating temperature of 191 °C. The crystal structure, surface morphology, chemical electron states, and optical characteristics were thoroughly examined using X-ray diffraction (XRD), scanning electron microscopy (SEM), transmission electron microscopy (TEM), X-ray photoelectron spectroscopy (XPS), and UV–visible diffuse reflectance spectroscopy (UV-Vis). To improve the gas adsorption capacity and sensor response effectiveness, this study examined how Co-doping and UV excitation modified the lattice parameter, specific surface area, band gap energy, and photoluminescence properties of MOF-ZnO materials. Additionally, the gas-sensing mechanism was elucidated, providing both experimental data and theoretical insights to facilitate the development of novel high-performance gas sensors. To achieve the creation of gas sensors with lower detection limits and faster reaction times, our future research will concentrate on further optimizing the material preparation and modification processes.

## Figures and Tables

**Table 1:** Lattice parameters, average grain sizes, and Full Width at Half Maximum (FWHM) values of pure MOF-ZnO and samples with varying Co-doping levels.

Sample	Lattice Parameter a = b (Å)	Lattice Parameter c (Å)	Grain Size (nm)	FWHM (°)
pure MOF-ZnO	3.2518	5.2406	7.06	1.17
1-Co/MOF-ZnO	3.2475	5.2159	6.81	1.21
3-Co/MOF-ZnO	3.2436	5.198	6.66	1.24
5-Co/MOF-ZnO	3.2519	5.1796	8.09	1.02
7-Co/MOF-ZnO	3.2532	5.1668	9.11	0.91

Based on (101) plane.

**Table 2:** Responses of several ZnO-based gas sensors to VOCs.

Sample	Gas	Concentration (ppm)	Working Temperature (°C)	Response (Ra/Rg)	Light	Refs.
In_2_O_3_/ZnO	formaldehyde	100	300	53.20	/	[44]
Pt-ZnO	triethylamine	100	200	242.00	/	[45]
ZnO/Ti_3_C_2_Tx MXene	triethylamine	100	160	28.20	/	[46]
N_3_-loaded ZnO nanocluster	ethyl alcohol	200	225	75.10	Green	[18]
SnO_2_-ZnO aerogels	ethyl alcohol	100	300	15.50	UV	[47]
ZnO-Ni nanotetrapods	n-butanol	100	400	186.00	/	[27]
5-Co/MOF-ZnO	n-butanol	100	191	1259.06	UV	This work

## Data Availability

Data are contained within the article.
